# Using scenario-based training to promote information literacy among on-call consultant pediatricians

**DOI:** 10.5195/jmla.2017.79

**Published:** 2017-07-01

**Authors:** Jonas Pettersson, Emil Bjorkander, Sirpa Bark, Daniel Holmgren, Per Wekell

## Abstract

**Background:**

Traditionally, teaching hospital staff to search for medical information relies heavily on educator-defined search methods. In contrast, the authors describe our experiences using real-time scenarios to teach on-call consultant pediatricians information literacy skills as part of a two-year continuing professional development program.

**Case Presentation:**

Two information-searching workshops were held at Sahlgrenska University Hospital in Gothenburg, Sweden. During the workshops, pediatricians were presented with medical scenarios that were closely related to their clinical practice. Participants were initially encouraged to solve the problems using their own preferred search methods, followed by group discussions led by clinical educators and a medical librarian in which search problems were identified and overcome. The workshops were evaluated using questionnaires to assess participant satisfaction and the extent to which participants intended to implement changes in their clinical practice and reported actual change.

**Conclusions:**

A scenario-based approach to teaching clinicians how to search for medical information is an attractive alternative to traditional lectures. The relevance of such an approach was supported by a high level of participant engagement during the workshops and high scores for participant satisfaction, intended changes to clinical practice, and reported benefits in actual clinical practice.

## BACKGROUND

The digital revolution has transformed the field of medicine, resulting in a situation where good clinical practice depends not only on traditional clinical competence, but also on the clinician’s information literacy [1]. However, making digital search tools available to medical staff does not guarantee that they are widely [2] and efficiently used, and several studies show that information-searching skills among medical doctors are not as widespread as one might expect [3–5]. It is also known that many medical students rely on second-rate sources for medical information [6]. To safeguard good clinical practice, it is, therefore, highly relevant to address the issue of how to improve competence in information searching and assessment of information reliability among clinicians.

Information-searching skills are often taught to clinicians through lectures using defined search methods without taking into account clinical relevance and adult learning principles [7–9]. By contrast, a scenario-based approach has the potential to address key aspects of adult learning by encouraging active participation in case discussions and improving relevance by taking clinical situations as a starting point [10]. This type of approach for promoting information literacy among clinicians has rarely been addressed in the literature [11].

In 2013, medical librarians in the Västra Götaland region were approached for assistance in developing and implementing a learning module targeting information search skills as part of a continuing professional development (CPD) program for on-call consultant pediatricians. The two year-long CPD program was introduced in western Sweden in 2010, with objectives developed from a needs assessment that had been carried out in the target group [12, 13]. The overall aim of the CPD program—which utilized preparatory reading assignments, real-time scenario training, and reflection before returning to clinical practice to apply the new skills—was to enable participants to work independently as on-call consultant pediatricians [14, 15]. The objective of the information searching module was, therefore, formulated as follows: “Upon completion of the learning module, you should be able to solve clinical problems by means of information searches and assess the reliability of medical information on the web.” We decided that the information-searching module would be based on real-time scenarios and that the learners would first be allowed to independently search for medical information in their own preferred ways before educators intervened to help overcome any problems encountered [11].

## STUDY PURPOSE

This case study describes the authors’ experiences of using real-time scenarios to teach information literacy skills to on-call consultant pediatricians.

## CASE PRESENTATION

### Information-searching workshops

The workshops took place at Sahlgrenska University Hospital on May 8, 2014 (twenty-one participants), and March 3, 2016 (fourteen participants). Of the total of thirty-five participants, fifteen were men and twenty were women. Four of the participants held or had recently held appointments as department heads or similar. Eleven participants worked in university hospitals, and twenty-four participants worked in secondary-level hospitals. The participants represented a wide range of different pediatric subspecialties. Most had significant experience working as on-call consultants, in some cases for decades.

Twelve different real-time medical scenarios (i.e., scenarios that used the same time frames as the real events that the scenarios emulated) were designed to stimulate the use of different approaches to find relevant medical information (Table 1). The scenarios were developed and informed by a pre-course needs assessment carried out in the target group. Since our team of educators included both experienced pediatricians and a medical librarian, it had a unique body of competencies to develop scenarios resembling actual challenges faced by on-call consultant pediatricians. Also, we crafted the scenarios such that the participants needed to utilize a variety of search strategies and tools.

**Table 1:** Examples of scenarios used during the workshop

**Scenario 1**	

Clinical situation	Johan, two years old with Kawasaki syndrome, is being cared for in the pediatric ward. You are sure about the diagnosis, but Johan has not responded to standard treatment with intravenous immunoglobulin (IVIG) in combination with acetylsalicylic acid (ASA). 48 hours after IVIG, Johan still has a fever of 39°C, rising C-reactive protein at 211 mg/L, and increased absolute neutrophil count at 18×109/L. You want to intensify the treatment and are considering what alternative you ought to choose.
Questions for information search	What are treatment alternatives? What is the scientific support for different treatment alternatives? Which alternative do you choose, and how do you explain your decision?
Questions for large group discussions	Are you satisfied with the information you found? Where did you start your search? Which search words did you use? Where did you find the most relevant information?

**Scenario 2**	

Clinical situation	During the night, Adam, five years old with a terminal illness, has been admitted to the ward after being cared for by his parents at home. The parents arrived at the ward with Adam after he had a seizure at home that got out of control. In the morning, the nurse said that she didn’t think Adam would live much longer. The family is Muslim (Christian) and you are Christian (Muslim). You realize that you lack sufficient knowledge about what wishes a Muslim (Christian) family could have regarding the care of Adam before and after his death.
Questions for information search	Consider how you can improve your knowledge through a literature search to be better prepared for counseling the parents. After the literature search, write down five keywords that summarize the most important knowledge that you gained. What have you noted down?
Questions for large group discussions	How trustworthy do you consider the information you found? How do you evaluate the trustworthiness of the information you found? What criteria did you use for this evaluation?

**Scenario 3**	

Clinical situation	You receive a telephone call from the junior doctor on-call, as he wants to ask you about Sven, aged six months, who has been admitted to the ward with rib fractures. You are, of course, worried about the situation and how the fractures may have occurred. The father says that Sven is adopted from Ethiopia, came to Sweden two months ago, and has been treated for rickets due to vitamin D deficiency since one week of age. The father explains that Sven’s rib fractures are caused by his vitamin D deficiency.
Questions for information search	Use the computer to find out if there is any substance to the father’s explanation: Can vitamin D deficiency contribute to rib fractures? How does the information you find influence your assessment of the case?
Questions for large group discussions	What information source or sources did you build your assessment on? Did you consider it necessary to consult more than one source in this case?

Scenario 1 required participants to find treatment alternatives for a given diagnosis and to evaluate these alternatives based on scientific evidence. The participants were, therefore, asked to employ a search strategy that focused on rapidly finding options backed by scientific evidence and to explain their choices in the ensuing discussion. In scenario 2, we asked participants to find information about funeral traditions that were foreign to their own customs, which was a broader question that could include a multitude of potential search strategies, including Google searches. In scenario 3, participants were asked to evaluate whether a specific injury was caused by the implied medical condition or might instead be the result of domestic abuse. This problem formulation called for a search strategy with a focus on a specific diagnosis.

Approximately three weeks before the workshop, participants were sent an email with a reading assignment with literature on medical search tools, including information on BMJ Best Practice, the Cochrane Library, UpToDate, PubMed, and other search options. The participants were also told to bring any portable devices they liked (e.g., smartphones or tablets) to use in their role as an on-call consultant pediatrician.

On the day of the workshop, the participants gathered in a computer hall and were encouraged to sit separately or in small groups, depending on how they preferred to work. The context of the workshop was given as follows: “In the scenarios, you will be faced by clinical cases you encounter during rounds or as reported to you in your home or in the hospital by the junior doctor on call. You have 10–15 minutes to find relevant information and move the case (situation) forward. You are allowed and encouraged to use any means you find appropriate to find the answers you need.”

The participants were then shown each specific clinical scenario with associated questions on a screen at the front of the computer hall for the duration of the time that the participants needed to accomplish the task. This was followed by a five-minute discussion on the specifics of the clinical problem, headed by one of the pediatricians in the educator team. The medical librarian then led a longer discussion concerning different search strategies, tools, tips, and troubleshooting of specific problems encountered as well as the reliability of different information sources. The starting points for the discussions were formulated beforehand (Table 1). The participants were encouraged to share their own strategies and tips with the group. The discussions typically lasted fifteen to twenty minutes, and a whole scenario, thus, usually took thirty to forty minutes to complete. The workshop was scheduled for four hours and included as many of the twelve scenarios as could be completed during the assigned time.

### Participant learning outcomes

Learning outcomes were evaluated from three aspects corresponding to Kirkpatrick levels 1–3: reaction, learning, and behavior [16]. After the 2014 and 2016 workshops, participants’ satisfaction with the learning module was evaluated in accordance with Kirkpatrick level 1. After the 2016 workshop, participants’ intentions to make changes to their clinical practice and reported benefits to their clinical practice were also evaluated in accordance with Kirkpatrick levels 2 and 3.

#### Satisfaction with the workshop (reaction)

In response to the question, “How do you assess the workshop as a whole, on a scale from 1 (very bad) to 6 (very good)?,” participants gave a mean score of 5.2 (range, 4–6) in 2014 and 5.3 (range, 4–6) in 2016. In response to the question, “Would you recommend this course to a colleague in a situation similar to yours, on a scale from 1 (not at all) to 6 (yes, definitely)?,” participants gave a mean score of 5.2 (range, 4–6) in 2014 and 5.4 (range, 4–6) in 2016.

#### Intention to change clinical practice (learning)

Immediately after the 2016 workshop, an additional questionnaire was used to evaluate participants’ intention to make changes to their clinical practice. Participants were asked, “Do you think that today’s workshop about searching for information will change the way you work as an on-call consultant pediatrician in the future?” Of the fourteen participants who answered the question, thirteen said that the workshop would change the way they intended to work in the future.

#### Reported change in clinical practice (behavior)

One month after the 2016 workshop, participants were given a third questionnaire evaluating benefits to their clinical practice. Participants were asked, “Have you, in your clinical work, benefited from the workshop on information searching?” Of the twelve participants who answered the question, nine stated that the workshop had benefited them in their clinical work.

### Reflections on the workshop by educators

During the workshops, we experienced a high level of engagement and active contributions from the participants. For example, during the first workshop, the participants ignored the scheduled coffee break because they desired more time for searches and discussions.

The discussions brought up many tips, originating both from the pediatricians and the medical librarian, about how and where to search for different types of information. Discussions ranged from the correct use of quotation marks when carrying out a phrase search in databases like PubMed to more overarching topics such as how search engines like Google filter results, affecting searches performed by medical staff as well as those carried out by patients.

The medical librarian also found that the workshops were a good way to attain information on the kinds of problems that clinicians encountered as well as what strategies and skills were applied to overcome those problems. For example, the lack of a refined image search in any of the various dermatology databases that were accessible during the workshops forced the clinicians to use Google image search to solve a specific scenario. The workshops were also useful for identifying skills that clinicians might need to assess clinical problems swiftly and with as few errors as possible. The clinician educators found that the workshop improved their own search skills in a way that could be useful in other educational contexts.

Finally, in 2016, several participants specifically emphasized the value of the workshop when it was evaluated as a whole using open, reflective questions.

## DISCUSSION

Instead of creating a preformulated lecture, we developed workshops in which participants were asked to solve medical scenarios with help from each other and support from educators [8, 17, 18]. As a consequence of the clinical relevance of the scenarios, there was a shared sense of purpose and urgency in the room that promoted learning in a broad sense [19, 20].

Framed by the scenario at hand and based on what was brought up by the participants, the workshops could take many directions, from solving very specific technical difficulties to broad overarching discussions [15, 21]. We ensured that relevant topics were covered by giving careful attention to designing and formulating the scenarios. Each scenario was different and designed to stimulate the participants to use a wide array of search strategies and tools. Furthermore, it was important to consider the sequence of the scenarios, as not all could be completed during a typical workshop. It is, therefore, important to have the first scenarios in a workshop cover as much of the relevant training as possible.

In the workshops, participants started out by performing searches in their own preferred ways, a strategy that potentially benefited the workshop in several ways [15]. First, participants learned how to improve their existing skills by identifying new strategies that they themselves deemed relevant for solving the scenarios. Second, participants were able to learn from each other during the workshop [22]. Third, we gained knowledge about how clinicians search for medical information in real life, as opposed to how they describe their search practices in surveys.

One challenge was related to computer use and the overall information technology structure of the hospital. Many participants had problems accessing different websites and were hampered by login procedures. Because one of the purposes of the workshop was to enhance search skills both in and outside the hospital setting, this was not a completely negative experience. Computer-related problems initiated discussions about technical issues, such as how to log in to information resources at the hospital and from home, which are important aspects of information retrieval that a majority of participants did not fully recognize [23].

Educators who deal with problems as they arise or are expected to have knowledge about a variety of topics that come up in discussion can sometimes experience their role as challenging and even daunting, especially in comparison to giving a formal lecture, which can be prepared in advance and rehearsed [21]. This highlights the importance of practicing in a safe environment, not only for participants, but for educators as well. In the present workshops, this was achieved by the close, collaborative work between pediatricians and librarians in developing and implementing the learning module from the very beginning. This issue, however, will be further addressed by organizing seminars for educators in the future in which they can practice their educational skills as a team.

We believe that a scenario-based approach is an attractive alternative to traditional lectures on educator-defined topics. The experiences of our multidisciplinary team of educators illustrate that we are educators and learners at the same time. Even though the present approach to learning can be daunting, it ensures that we do not deliver an outdated lecture on a tool that is no longer used; rather, this approach keeps us, by its very format, updated on the latest developments in our field.
